# A Versatile Optoelectronic Tweezer System for Micro-Objects Manipulation: Transportation, Patterning, Sorting, Rotating and Storage

**DOI:** 10.3390/mi12030271

**Published:** 2021-03-06

**Authors:** Shuzhang Liang, Yuqing Cao, Yuguo Dai, Fenghui Wang, Xue Bai, Bin Song, Chaonan Zhang, Chunyuan Gan, Fumihito Arai, Lin Feng

**Affiliations:** 1 School of Mechanical Engineering & Automation, Beihang University, Beijing 100191, China; liangsz13nq@buaa.edu.cn (S.L.); caoyuqing@buaa.edu.cn (Y.C.); daiyuguo5612@163.com (Y.D.); xuebai@buaa.edu.cn (X.B.); songb@buaa.edu.cn (B.S.); zhangcn@buaa.edu.cn (C.Z.); chunyuangan@126.com (C.G.); 2 BEIGE Institue of Robot & Intelligent Manufacturing, Weifang 261000, China; weiner.wang@gbirobot.com; 3 Department of Mechanical Engineering, University of Tokyo, Tokyo 113-8656, Japan; arai-fumihito@g.ecc.u-tokyo.ac.jp; 4 Beijing Advanced Innovation Center for Biomedical Engineering, Beihang University, Beijing 100083, China

**Keywords:** microparticle manipulation, optoelectronic tweezers, sorting, rotating

## Abstract

Non-contact manipulation technology has a wide range of applications in the manipulation and fabrication of micro/nanomaterials. However, the manipulation devices are often complex, operated only by professionals, and limited by a single manipulation function. Here, we propose a simple versatile optoelectronic tweezer (OET) system that can be easily controlled for manipulating microparticles with different sizes. In this work, we designed and established an optoelectronic tweezer manipulation system. The OET system could be used to manipulate particles with a wide range of sizes from 2 μm to 150 μm. The system could also manipulate micro-objects of different dimensions like 1D spherical polystyrene microspheres, 2D rod-shaped euglena gracilis, and 3D spiral microspirulina. Optical microscopic patterns for trapping, storing, parallel transporting, and patterning microparticles were designed for versatile manipulation. The sorting, rotation, and assembly of single particles in a given region were experimentally demonstrated. In addition, temperatures measured under different objective lenses indicate that the system does not generate excessive heat to damage bioparticles. The non-contact versatile manipulation reduces operating process and contamination. In future work, the simple optoelectronic tweezers system can be used to control non-contaminated cell interaction and micro-nano manipulation.

## 1. Introduction

The observation and manipulation of objects at the micro and nano scales is also one of the core technologies to study the micro/nano-science [1]. Accurate, rapid, and non-destructive manipulation of micro/nanoparticles is the basis for a range of applications such as biomedicine [2,3] and micro/nano-fabrication [4,5,6]. A number of micro-/nano-scaled methods have been proposed to manipulate [7,8,9] and fabricate micro/nanomaterials [10], including acoustic [11], optical tweezers [12,13], electrokinetic [14], magnetic [15,16,17] tweezers, and microrobot-assisted [18]. Although these methods are very mature technologies with excellent precision and diversity for micromanipulation, they have potential shortcomings that limit their use in certain applications. For example, optical tweezer technology requires high light power, which can cause physiological damage to trapped cells. Dielectrophoresis (DEP) technology requires complex electrodes and a long production process. In addition, in previous work, the microelectrode lacked flexibility and could not provide real-time variable electrode patterns to meet different manipulation requirements.

Optoelectronic Tweezers [19,20], a new manipulation technology that combines optical tweezers and dielectrophoresis, provides a more versatile approach for micro-/nano-scale manipulation [21] and fabrication [22,23,24]. OET uses light to control DEP forces for manipulation instead of direct manipulation by photon-generated pressure, and it does not require large power for generating capturing force. Thus, it can reduce the heat to avoid damaging cells and other bioparticles. Moreover, OET systems use a fixed sandwich-like electrode that achieves excellent manipulation precision by using a projected micro-pattern on the chip. Projected micro-pattern provides the system with a flexible electrode that is not available in the predesigned structures used in conventional DEP systems. OET has been widely used in the manipulation of micro-objects [25] such as cell culture [26,27,28,29] and microrobot [30,31], and the assembly of sub-millimeter electronic [32,33,34] and electrical devices [35,36]. For example, OET is also used to analyze the relative hardness of red blood cells [37] and conditions that affect the cell membranes of swimming Enterobacter aerogenes bacteria [38]. All of these show that OET has a wide range of applications in the manipulation of micro-nano particles and cells [39,40,41]. However, the conventional OET system, designed for a single function, cannot provide real-time variable electrode patterns on the same chip to meet the requirements of diverse manipulation. Besides, it is difficult to manipulate particles with complex dimensions or a wide range of sizes in the OET system.

**Figure 1:**
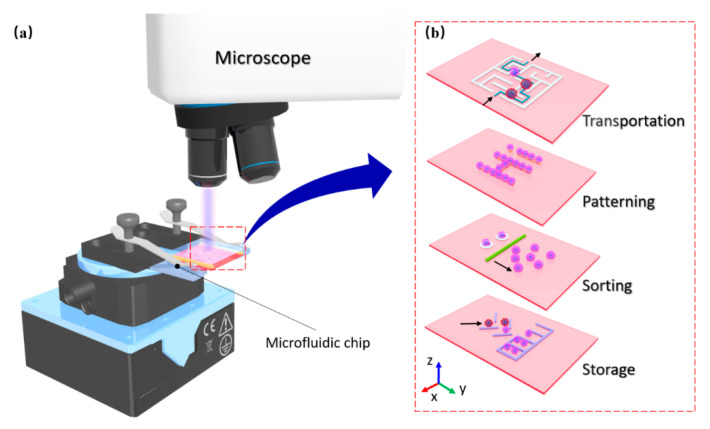
Conceptual overview of manipulation of microparticles via OET. (**a**) Overview of on-chip manipulation system; (**b**) Diverse manipulation of microparticles by light pattern: transportation, patterning, sorting and storage.

In this paper, a simple optoelectronic tweezer system was designed and established as a platform for controlling micro-objects that could accurately control cells and particles with different scales. In the OET system, particles of different sizes were trapped in an optical image, and the electrodes formed an inhomogeneous electric field. And versatile micromanipulation was achieved by moving the light image in the same microfluidic chip, as shown in Figure 1. Meanwhile, the light image rotated the particles. In this way, there was no need to worry about fabricating microrobots or performing special pretreatments on the microparticles. In addition, in case of low light power, there was no damage to the *Euglena gracilis* such as to membrane in the entire experiment.

## 2. Theory and Methods

### 2.1. Dielectrophoresis Force Analysis

Generally, in OET, the photovoltaic electric field and the current density generated in the material due to illumination can be calculated with the well-known photorefractive effect [42]. These equations assume one single type of free charge carrier and a single impurity center, having concentrations *n* and *N* respectively, which is a good approximation under moderate light intensities. Thus, the rate equations governing the evolution of the system under arbitrary illumination *I*(*r*,*t*) are given by:

(1) $$\frac{\partial n}{\partial t}=sIN_{D}-\gamma nN_{A}-\frac{1}{q}\vec{\nabla }\vec{J}$$

(2) $$\frac{\partial N_{D}}{\partial t}=-\frac{\partial N_{A}}{\partial t}=-sIN_{D}+\gamma nN_{A}$$

(3) $$\vec{J}=q\mu n\vec{E}-qD{\vec{\nabla }}_{n}+qsIN_{D}L_{p}{\vec{\nabla }}_{p}$$

where *N_D_* and *N_A_* are the donors and acceptors concentrations respectively. *s* is the photoionization cross-section. γ is the recombination constant. *J* is the current density. μ is the mobility. *E* is the total electric field. *D* = μ*k_B_T*/*q* is the diffusion coefficient. *L_p_* is the photovoltaic transport length and ${\vec{\nabla }}_{p}$ is the unit vector in the direction of the polar axis.

Then, when a suspended sphere particle was exposed to an electric field, electrical charges were generated on its interface with the medium, resulting in the formation of dipoles inside the particle. When an AC frequency electrical field of amplitude *E* is applied to a cell suspension, each particle, of radius *a*, is polarized by the external field and is equivalent to a dipole with moment given by:

(4) $$p=\alpha E=4\pi \varepsilon_{1}R^{3}\frac{\varepsilon_{2}^{*}-\varepsilon_{1}^{*}}{\varepsilon_{2}^{*}+2\varepsilon_{1}^{*}}E=4\pi \varepsilon_{L}R^{3}K\left(\omega \right)E$$

where α is the polarizability, $\varepsilon_{2}^{*},\;\varepsilon_{1}^{*}$ are the complex permittivity of the medium and particles respectively. To model a biological cell, a membrane-covered sphere must be considered. It results from the dielectric properties of the cell and depends on the particle’s size and the frequency and amplitude of the applied field. The time-averaged force on a dipole due to a non-uniform alternating electrical field can be calculated as:

(5) $$\bar{F}=\frac{1}{2}Re\left[\left(p\nabla E^{*}\right)\right]$$

where $E^{*}$ denotes the complex conjugate of *E* and *Re* the real part of the complex expression. The time-averaged DEP force applied on a spherical particle is expressed as [43]:

(6) $$F=2\pi R^{3}\varepsilon_{m}Re\left[K\left(\omega \right)\right]\nabla E_{rms}^{2}$$

where $\varepsilon_{m}$ is the permittivity of the medium, *R* is the radius of the particle, Re[K(ω)] is the real part of Clausius-Mossotti factor, and $E_{rms}$ is the root-mean-square (rms) value of the applied electric field. K(ω) depends on the dielectric, geometrical, and polarizability properties of the particle and the suspending medium and is expressed as:

(7) $$K\left(\omega \right)=\frac{\varepsilon_{p}^{*}-\varepsilon_{m}^{*}}{\varepsilon_{p}^{*}+2\varepsilon_{m}^{*}}$$

where $\varepsilon_{p}^{*}$ and $\varepsilon_{m}^{*}$ are the complex permittivity of the particle and the suspending medium, respectively, which are expressed as:

(8) $$\varepsilon_{p}^{*}=\varepsilon_{p}-j\frac{\sigma_{p}}{\omega }$$

(9) $$\varepsilon_{m}^{*}=\varepsilon_{m}-j\frac{\sigma_{m}}{\omega }$$

where $\sigma_{p}$ is the electric conductivity of the particle, $\sigma_{m}$ is the electric conductivity of the medium, ω is the angular frequency of the applied alternating current (AC) signal, and *j* is the imaginary unit.

(10) $$F_{resistence}=C_{D}A\frac{\rho u^{2}}{2}$$

where $A=\pi R^{2}$ represents the cross-sectional area of the particle, R is its diameter, u is the moving speed, ρ is the density of the solution, and $C_{D}$ is the drag coefficient, which is related to the diameter of the cell, and can be expressed as:

(11) $$C_{D}=\frac{12\mu }{\rho Ru}$$

where μ is the kinematic viscosity of the liquid. The maximum of $F_{resistence}$ equals to *F*.

Besides, for micromanipulation, the Brownian motion of a particle is a result of the thermal motion of the molecular agitation of the liquid medium. Much stronger random displacement of a particle is usually observed in a less viscous liquid, smaller particle size, and higher temperature. However, for particles of diameter over 1 μm, sedimentation is responsible for the displacement of particles [44]. Hence, Brownian motion is negligible here for particles of size 1 μm and above. For our experiment system, the size of all the particles is larger than 1 μm. Thus, it does not show a remarkable Brownian motion.

Therefore, using the expressions for hydrodynamic and dielectrophoretic forces, the transient motion of a particle can be rewritten as:

(12) $$m_{p}\frac{d{\bar{u}}_{p}}{dt}={\bar{F}}_{d}+{\bar{F}}_{DEP}$$

(13) $$I_{p}\frac{d\omega_{p}}{dt}=\int \left(x_{s}-x_{p}\right)\times \left[\left(T_{H}+T_{M}\right)\cdot n\right]dS$$

where ${\bar{u}}_{p}$ and $\omega_{p}$ are, respectively, the translational velocity and rotational velocity of the particle. $x_{s}$ and $x_{p}$ are, respectively, the position vector of the surface and center of the particle. The hydrodynamic force, ${\bar{F}}_{d}$, and the time-averaged AC DEP force,$\;{\bar{F}}_{DEP}$, acting on the particle are obtained, respectively, by integrating the hydrodynamic stress tensor, *T_H_*, and the time-averaged Maxwell stress tensor, *T_M_*, over the surface of the particle. Where *n* is the unit normal vector on the corresponding boundary. Where $m_{p}$ and $I_{p}$ are, respectively, the mass and moment of inertia of the particle. Accordingly, the position and orientation of the particle can be obtained by solving the following equations:

(14) $$\frac{dx_{p}}{dt}={\bar{u}}_{p}$$

(15) $$\frac{d\theta_{p}}{dt}=\omega_{p}$$

Besides, the particle motion alters the surrounding flow field and electric field, which in turn affects the hydrodynamic and DEP forces and their corresponding torques acting on the particles. According to these formulations and combined with the observation of the CCD camera, we could manipulate the particles in real-time.

According to Equations (6) and (7), the DEP force is proportional to the volume of the particle and the square (rms) value of the applied electric field gradient. Moreover, it is related to the permittivity of the particle and the suspending medium. Nevertheless, the direction of the vortex only depends on Re[K(ω)]—the real part of the Clausius-Mossotti factor, at which Re[K(ω)] is greater than zero and the particles undergo a positive DEP to move towards the regions with large electric-field gradient. Otherwise, the DEP force repels the particle. Thus, diverse manipulation is achieved by using different optical projection patterns to generate different dielectrophoretic forces in the same microfluidic chip.

### 2.2. Simulation of Electric Field Distribution

**Figure 2:**
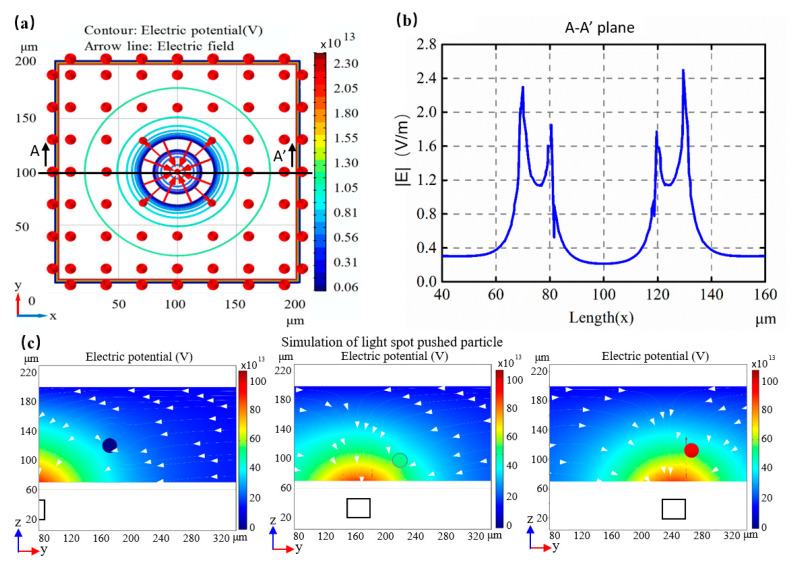
The distribution of electric field in the OET. (**a**) The electric potential and the directional distribution of the electric field; (**b**) The strength of electric fields in different lengths. A peak value appears near the junction of brightness and darkness, and then two peaks appear in the trapped ring; (**c**) Simulation of particles being pushed by light spot.

The COMSOL Multiphysics 5.5a (COMSOL Inc. Stockholm, Sweden) package was used to simulate the distribution of the electric field within an optoelectronic chip to analyze its internal dielectrophoresis forces. A three-dimensional ring-shaped virtual electrode was constructed so that the electrode boundaries satisfied the Dirichlet boundary condition, *V* = *V*_0_, while the remaining boundary conditions satisfied the Neumann border condition, ∂*V*/(∂*n* = 0). A theoretical light-induced dielectrophoresis model was then applied to obtain the distribution of the electric field by applying a sine wave-shaped source-applied voltage of 10 V_pp_ at 100 kHz through a liquid with a conductivity of 0.2 mS/m and permittivity of 78. Figure 2 shows the resulting distribution of electric field across different dielectric lengths. Figure 2a shows the electric potential and directional distribution of the electric field. According to Figure 2b, a potential trap was formed in a region of low electric field intensity at the center of the trapped ring where a peak was formed near the bright-dark junction and two peaks were formed in the trapped ring. Thus, the trapped model was composed of two strong electric field regions set apart from the center of the electrode and a weak region located at the center. When the nDEP particles moved away from the central streamline, the pDEP particles were aggregated along it. According to the simulation results shown in Figure 2 and Equation (6), the magnitude of the DEP force could be estimated within a range from several pN to several tens of pN and was therefore capable of generating forces sufficient to move the particles in a manner consistent with other reported results [19,45]. In addition, the process of particle being pushed by the light spot was simulated by software. The electric field was based on the application of a sine wave-shaped source-applied voltage of 10 V_pp_ at 100 kHz. The conductivity and permittivity of medium were 0.2 mS/m and 78, respectively, and those of of particle were 0.23 mS/m and 2.56, respectively. The result showed that light could push the particle forward without exceeding its maximum velocity, as shown in Figure 2c. If the moving velocity of the light exceeded its maximum, the particle would skip over the light spot (Supplementary Material Video S1).

## 3. Experiments and Results

### 3.1. Fabrication of the Microfluidic Chip and System Setup

**Figure 3:**
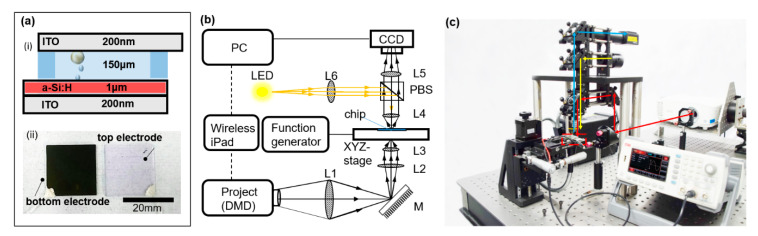
Schematic diagram of OET system installed on a microfluidic chip. (**a**) Structure of fabricated a-Si:H microfluidic chip. (**b**) Image projection path through lenses L1, L2, and L3 and reflection from mirror M. L4, PBS, L5, and CCD comprise the observation path; LED, L6, PBS, and L4 comprise the illumination path. (**c**) Picture of the OET system.

The microfluidic chip was established based on the structure diagram in Figure 3a. The bottom electrode consisted of an a-Si:H film that fabricate on ITO glass by PECVD [25,30]. The top electrode was ITO glass. The ITO electrode was placed on the photoconductive electrode with a 150-μm-thick spacer using double-sided adhesive. The deionized water without surfactant is used as the medium. A sample droplet containing target microparticles was put in the spacer. The optoelectrofluidic chip, composed of a-Si:H layer, was connected to the Function Generator (UNI-T co., ltd., Shanghai, China) at both electrodes. The preliminary photoelectric conversion of a-Si:H film was carried out by projecting light into the chip. Thus, the uniform electric field generated in the light image was used to manipulate the microparticles.

The experimental platform was established based on the functional structure diagram in Figure 3b. The system was constructed using a Digital Micromirror Device (DMD) projector (BenQ E580, Taiwan, China) as a pattern generating device and a-Si coated on an indium tin oxide (ITO) electrode (NOZO. co., Shenzhen, China) as a photoconductive layer. The image space in the output of the projector was programmed by a computer. Then, the optical image contraction system consisted of two lenses with focal lengths (L1 and L2), a condense lens (L3), and a reflecting mirror (M). Subsequently, the image projected onto the chip and the real-time manipulation were formed and captured by a 20× objective lens (L4) and a charge-coupled device (CCD) camera (FLIR Systems Inc, Wilsonville, Oregon, USA), respectively. When necessary, the light source for illumination was provided by a Light Emitting Diode (LED) (Dahengguangdian co., Beijing, China) and concentrated as a straight beam through a focusing lens (L6). Finally, the signal generator was used to raise AC voltage up to 10 V_pp_ and a programmable computer image from the DMD-based projector was projected onto the photoconductive layer through a condenser lens (L3). Several electron-hole pairs significantly increased at the partially illuminated surface of the photoconductive layer. As a consequence, a non-uniform electric field with a virtual electrode resulted in electrokinetic phenomena including DEP. A picture of the OET system is given in Figure 3c.

**Figure 4:**
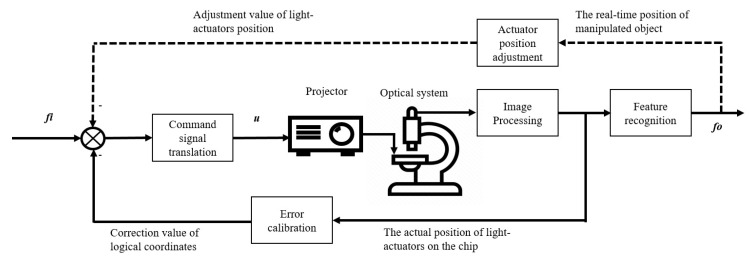
The schematic of image-based control system: input signal *f_i_* represents the logical coordinates of the light-actuators used to control the targeted object; output signal *f_o_* represents actual coordinates of targeted object on the chip.

The platform utilized an image-based, closed-loop control system to improve the operational precision and achieve partial auto-manipulation. The closed-loop control system contains the input signal *f_i_* that represents the logical coordinates of the light-actuators used to control the targeted object and the output signal *f_o_* that provides the actual coordinates on the chip of the targeted object, as shown in Figure 4. The control system applied two primary negative feedbacks. The first was an error calibration feedback designed to eliminate the difference between the coordinates of the objects in the logical and chip systems, as monitored by the CCD camera mounted on the microscope. The error introduced by hardware setup was systematic and could essentially be compensated through negative feedback calibration. The other feedback, which is shown by the dashed line in the figure, was used only in auto-manipulation mode, during which the light-actuator moved targeted particles along certain trajectories and its position was adjusted in real-time to match the movement of the targeted objects.

### 3.2. Different Size and Dimension of Microparticles Manipulation in OET

The manipulation capacity of OET was studied firstly. According to the characteristics of the particles, deionized water was selected as the medium. The permittivity and conductivity of deionized water were 78 and 2 × 10^−4^ S/m, respectively. To analyze the manipulation scale of microparticles, particles with different sizes were used for manipulation. Based on the size range of cells, the diameter of microparticles was 2 μm, 15 μm, 20 μm, 50 μm, 100 μm, and 150 μm, respectively. The external input signal was 10 V_pp_ bias voltage with an AC frequency of 100 kHz. The manipulating conditions of all microparticles remained the same. Finally, the light pattern was projected by a projector to manipulate the particles.

**Figure 5:**
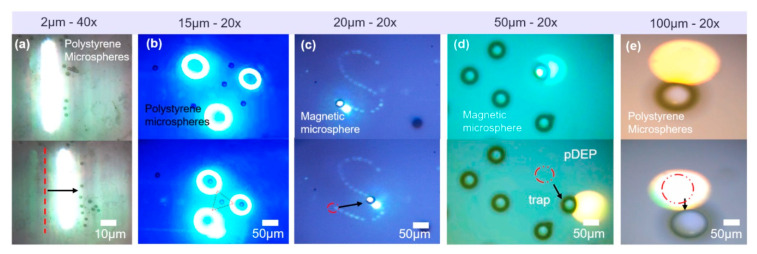
OET systems manipulate particles with different sizes. (**a**) 2 μm polystyrene microspheres; (**b**) 15 μm polystyrene microspheres; (**c**) 20 μm magnetic microspheres; (**d**) 50 μm magnetic microspheres; (**e**) 100 μm polystyrene microspheres.

**Figure 6:**
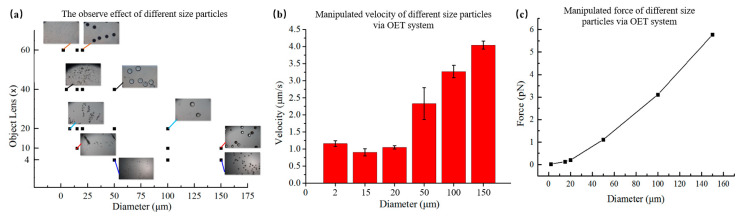
Manipulation velocity of particles with different sizes. (**a**) The observed effect of particles with different sizes through 4×, 10×, 20×, 40×, or 20× microscope objectives; (**b**) Manipulation velocity of particles with different sizes via OET system; (**c**) Manipulation force of particles with different sizes.

As shown in Figure 5, the polystyrene microspheres with a diameter of 2 μm were pushed by the light strip under the 40× objective lens. Then, the light circle also transported polystyrene microspheres with a diameter of 15 μm and trapped them in the center of the pattern under a 20× objective lens. Magnetic microparticles with a diameter of 20 μm and 50 μm were pulled by moving the light in the microfluidic chip. Finally, polystyrene microspheres with a diameter of 100 μm and 150 μm were repelled by the light under a 20× objective lens. The results showed that the optoelectronic tweezers platform had a large manipulated range of 2 μm~150 μm. In addition, in order to better observe particles with different sizes, they were observed in different multiples of the objective lens. The results are shown in Figure 6a. Since the maximum multiple objective lens used was 60× and was limited by the resolution accuracy of the system projection pattern, the minimum limit size of the particles that could be observed in this OET system was 1 μm. The minimum objective lens used in the system was 4×. Thus, the maximum limit size was 200 μm for manipulation. The manipulation velocity of particles with different sizes was analyzed by ImageJ software, as shown in Figure 6b. The results are consistent with the conclusion of Equations (6) and (10) that velocity increased with the increase of the diameter of the particle, and the maximum velocity was 4.04 μm/s. The polystyrene bead (diameter 150 μm) suffers a manipulated force of 5.77 pN. The manipulated force is calculated with Stokes’ law, as shown in Figure 6c. To prevent damaged on biological cell, we use a relatively low light power. Thus, the maximum velocity was only reach a few microns per second.

**Figure 7:**
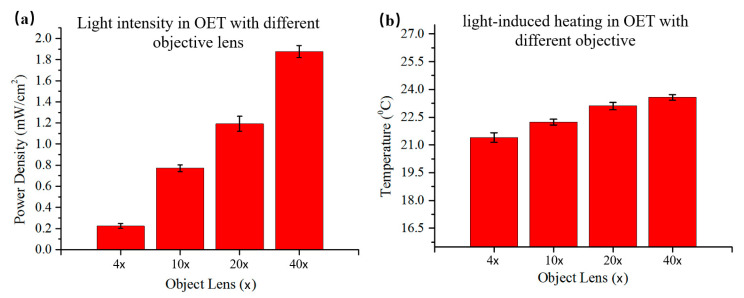
Light intensity and light-induced heating in OET with different objectives. (**a**) Optical power density measured with a square light pattern projected onto an OET device through 4×, 10×, 20×, 40×, or 60× microscope objectives; (**b**) Temperature measured with a square light pattern projected onto an OET device under 4×, 10×, 20×, 40×, or 60× microscope objectives.

Meanwhile, the light intensity of the illuminated light must be limited to prevent excessive dielectrophoresis force repelling the particles out of the observation field. Thus, the power density of the light pattern projected in the chip was measured by the optical power meter, as shown in Figure 7a. The density of optical power increased as the magnification of the objective increased, with the maximum power density measured to be 1.87 mW/cm^2^ under the 60× objective. On the one hand, the system is a bit open in order to simplify the structure of system, resulting in a loss of light density as the light propagates in its path. On the other hand, the light power of the project only used half of the maximum power (3000 Lm). Because if the optical power is too high, the OET system will reduce the resolution of the display. Thus, in the experiment, we used the light power is under a half of the maximum power of the projector. In addition, to study light-induced heating, a circle-shaped light pattern was projected onto the photoconductive layer of an OET device (without patterned photoconductive layer) filled with a buffer medium. The temperature profile of the illuminated region on the photoconductive layer was measured by using a temperature camera. As shown in Figure 7b, the temperature increased as light illumination accumulated. Moreover, the temperature of a large objective lens was higher than that of a small objective because the ability to concentrate light was stronger when the magnification of the objective lens increased. The whole temperature was below 37.5 °C in the OET system proposed in this paper, so it would not damage cells and other bioparticles. The heating effect described above might be moderate, but could be problematic when applied for extended periods to small volumes of fluid.

**Figure 8:**
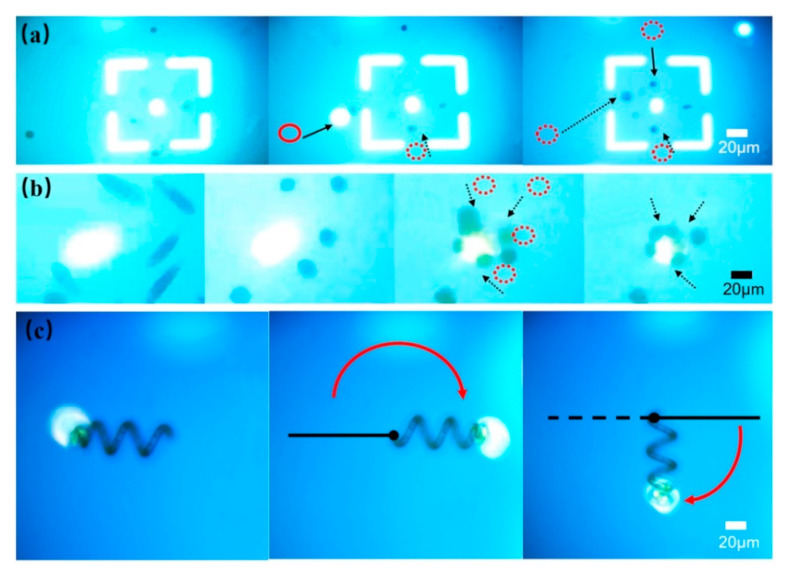
The manipulation of particles with different shapes particles via OET. (**a**) Manipulating macrophages at the square center in the 5% glucose solution; (**b**) Trapping *Euglena gracilis* in the deionized water; (**c**) Rotating the spirulina in the deionized water.

In addition, to study the manipulation effects of particles with different shapes in the OET system, one-dimensional sphere particles, two-dimensional rod-shaped particles, and three-dimensional spiral particles were used for manipulation. Firstly, macrophages with a diameter of about 20 μm were used as one-dimensional sphere particles for manipulation. 20 μL of macrophages were taken from the PBS culture medium and put into 1 mL glucose solution (the concentration was kept at 5% to maintain the osmotic pressure of the cells and avoid cell death). Macrophages then were injected into the microfluidic chip of the optoelectronic tweezers by a pipette. The external electric field conditions were: voltage 10 V_pp_ and AC frequency 100 kHz. The manipulation results indicated that macrophages were subjected to negative dielectrophoresis in a PBS and 5% glucose mixed solution. Therefore, cells were repelled to the square center by the light spot, as shown in Figure 8a.

Then, according to the scale range in previous work, the *Euglena gracilis* was used as a two-dimensional rod-shaped particle for manipulation due to its suitable size for manipulation in the OET system. Its diameter was about 15~30 μm and its length was about 70~100 μm. Moreover, *Euglena gracilis* is a multi-purpose phototrophic protist. *Euglena gracilis* has become a substitute microorganism for application-driven and commercial research of gene transformation as it contains abundant supplementary protein, vitamins, starch, and glucan. Meanwhile, *Euglena gracilis* have high tolerance to external pressures and extreme environmental conditions, such as acidic growth conditions and ionizing radiation. Therefore, *Euglena gracilis* was selected as the manipulated object of two-dimensional rod-shaped particles.

**Figure 9:**
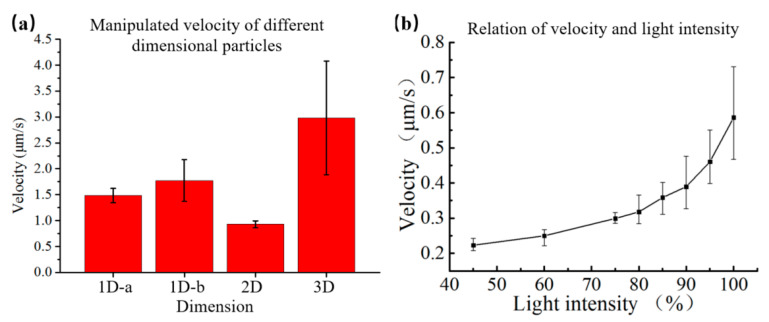
Manipulation velocity of particles with different shapes via OET. (**a**) Manipulation velocity of particles with different dimensions; (**b**) Relation of the velocity and input light intensity.

The external input signal was set as 10 V_pp_ voltage and 100 kHz AC frequency. The light spot was projected to assemble the *E. gracilis* in the deionized water, as shown in Figure 8b. The two-dimensional rod shape was rotated to be in parallel with the electric line under an electric field, resulting in a dipole as charge accumulated at both ends of the rod [46]. In the optoelectronic tweezers, the *E. gracilis* firstly overcame gravity and rotated to the vertical direction. Then it was attracted to the light spot. The results showed that the OET system could rotate and transport two-dimensional rod-shaped particles. The manipulation velocity of particles with different shapes via OET is shown in Figure 9a. By calculated with the Equation (10), the *E. gracilis* suffer a manipulated force of 1.13 pN.

Finally, natural microspirulina was used to demonstrate the manipulation of three-dimensional spiral particles in the OET system. On the one hand, spirulina has a natural helical structure with a large scale for observation. On the other hand, spirulina has been widely used in the preparation of nutritional products and special materials as well as drug delivery. Therefore, microspirulina was used as the manipulation object of three-dimensional spiral particles.

The body length of spirulina was 50~300 μm, the diameter was 20~30 μm, and the width of the line diameter was about 10 μm. After a certain number of sieves, spirulina with a suitable length was selected for manipulation. Subsequently, microspirulina was put into deionized water and injected into the microfluidic chip with a pipette. The external input signal of 10 V_pp_ bias voltage and 100 kHz AC frequency was applied. The microspirulina was rotated on the *XY*-plane by the light spot. The results indicated that the spirulina could be attracted and trapped by the light spot, as shown in Figure 8c. In addition, the manipulation velocity was controlled by adjusting the light intensity of the pattern, as shown in Figure 9b. When the light intensity is 3000 Lm, the maximum manipulation velocity was 0.6 μm/s, and the manipulation velocity decreased with the decrease of the light intensity. These results showed that the optoelectronic tweezers system could manipulate particles with different dimensions within a suitable scale range.

### 3.3. Versatile Manipulation for Transportation, Patterning, Sorting, and Rotating

**Figure 10:**
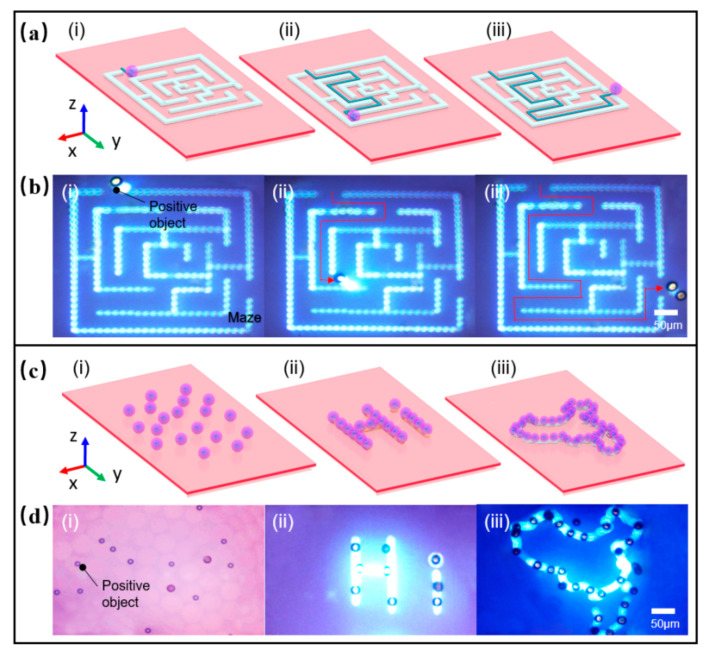
Transporting or patterning the microparticles via OET. (**a**) Schematic of transportation of microparticle through the maze; (**b**) Images of transportation of microparticle through the maze; (**c**) Schematic of patterning microparticle into different patterns; (**d**) Images of patterning microparticle of “Hi” word or map outline of China.

The transportation of microparticles is very important and fundamental for micromanipulation. OET could trap individual particles and then transport the particles to desired positions to further program and control their motion paths. As shown in Figure 10a, the planned path, formed by projecting the maze pattern on the microfluidic chip, was used to demonstrate the transportation of the magnetic microspheres (with a diameter of 20 μm). The external input signal of 10 V_pp_ bias voltage and 100 kHz AC frequency was applied. The magnetic microsphere was trapped at the center of the light spot and transported through the path of the maze, as shown in Figure 10b (Supplementary Material Video S2). The maximum manipulation velocity was 1.79 μm/s, and the manipulation force reach 0.34 pN.

In addition, some microelectrodes or microtissues were continuously and automatically fabricated and assembled by patterning. Figure 10c shows the different patterns that were used for the assembly of the particles. The external input signal of 10 V_pp_ bias voltage and 100 kHz AC frequency was applied. As shown in Figure 10d, the results illustrated the process of patterning magnetic microspheres with a diameter of 20 µm in a standard OET device (without micropatterns) to form a “Hi” word pattern and map outline of China (Supplementary Material Video S3). The pattern lasted for a short period of time after the light was turned off, but over time it gradually degraded and became unrecognizable. If the solution medium was UV-curable or heat-curable materials, it could be cured to form micropatterns structure with specific functions after the pattern was formed. Thus, it is of important significance in the preparation of micro-nano electrode devices.

**Figure 11:**
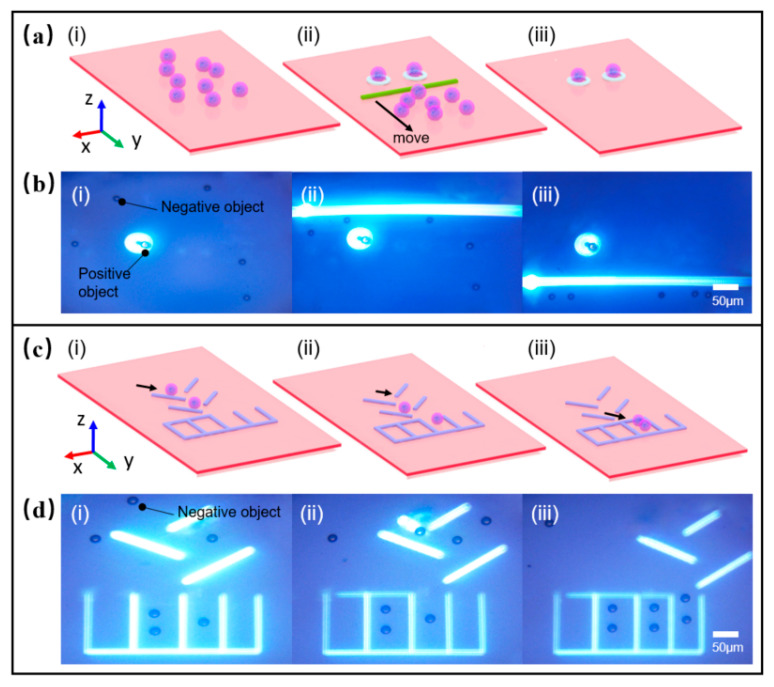
Sorting or storing the microparticles via OET. (**a**) Schematic of sorting microparticles via OET; (**b**) Images of sorting microparticles; (**c**) Schematic of storage microparticles; (**d**) Images of storage microparticles via OET.

In general, when the sample is pretreated, the non-target particles in the original sample should be sorted first. In the OET system, there was no need to sort different particles. The role of the optoelectronic tweezers system in sorting particles was verified, as shown in Figure 11a. Magnetic microspheres (20-μm-diameter) and polystyrene microspheres (15-μm-diameter) were mixed in deionized water at a ratio of 1:10. Then the solution was injected into the microfluidic chip. The applied external input signal was set to 10 V_pp_ bias voltage and 100 kHz AC frequency. The light circle was used to trap the magnetic microspheres and keep the particle in the original position. Then, the light line was used to move up and down the field of view. During the scan, the light line pushed polystyrene microspheres forward for sorting, as shown in Figure 11b (Supplementary Material Video S4). In the OET system, the magnetic microspheres left behind were affected by positive DEP force, while the polystyrene microspheres were affected by negative DEP force, which led to this separation phenomenon in the sorting experiment. As for the fact that fewer polystyrene particles remained behind the optical lines, one plausible explanation might be that these particles were pushed apart by the interaction forces between individual particles as they scanned the optical lines to drive the particles. As long as the optical line velocity did not exceed the maximum velocity of the normal particles, particles could be sorted. Here, the maximum velocity is 1.48 μm/s, and relative manipulated force is 0.21 pN. If the optical line velocity exceeded the maximum sorting velocity, all particles would be left behind the scanning optical line, making sorting impossible. The sorting function plays an important role in the separation and extraction of CTC circulating tumor cells and ascites cancer cells.

Under normal circumstances, in the microfluidic chip, a special zone is designed to store the particles or cells after sorting, transporting, or other manipulation such as interaction between cells. Thus, we created a stable light environment in the desired experimental zones on the chip to meet manipulating requirements, as shown in Figure 11c. The particles would not move into the storage zone on their own, whereas OET manipulation enabled them to move into the zone for storage or interaction. The optical linewidth and the gap between adjacent optical lines were set as 20 μm and 50 μm, respectively. The inclination angle for each virtual electrode was about 45°. As shown in Figure 11d, the particles with a diameter of 15 μm were moving along the virtual channel of the optical line-array. Then, the particles were transported into the storage groove, and the groove was sealed by a light line to prevent the particles from being repelled out of the groove (Supplementary Material Video S5).

**Figure 12:**
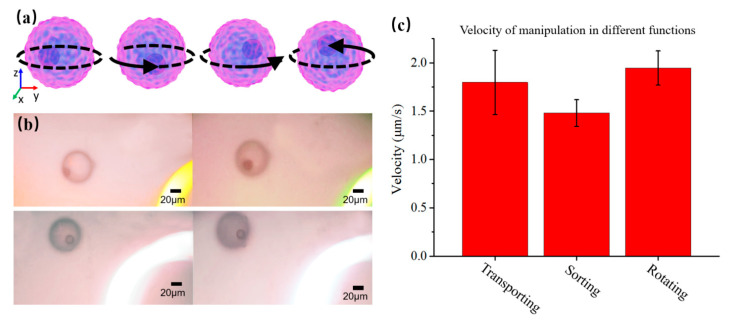
Rotating the microparticle via OET. (**a**) Schematic of rotating microparticle; (**b**) Images of rotating microparticle in the buffer medium; (**c**) Velocity of manipulation in different functions.

In addition, It is important to observe the structure of the particle from different places. Here, the OET system was used to rotate the particle for observation in the buffer medium, as shown in Figure 12a. The polystyrene microsphere solution, diluted with deionized water, was injected into the microfluidic chip. The external input signal of 10 V_pp_ bias voltage and 100 kHz AC frequency was applied. By pushing the particle from one side through the pattern of light, the particle was rotated because it was subjected to a non-uniform electric field force, as shown in Figure 12b (Supplementary Material Video S6). The rotating angular velocity was about 0.039 rad/s. The result showed that the particle could be rotated for 360° on the bottom plane along with the vertical *z*-axis. In contrast to most photoelectric tweezers that can only achieve translational movement of particles, we achieve rotational manipulation of particles that can be used to observe the overall properties of particles in 3D space. Figure 12c shows the results of manipulation velocity under different functions by OET.

## 4. Conclusions

In this work, we designed and established an optoelectronic tweezer system for versatile manipulation. Firstly, through simulation, we analyzed the distribution of the light-induced electric field used for manipulation. According to the simulation results, the magnitude of DEP force magnitudes could be estimated to be within a range from several pN to several tens of pN. Then, since the maximum and minimum multiple objective lens used was 60× and 4×, respectively, the minimum and maximum limit size of the particles that could be observed was 1 μm and 200 μm in the proposed OET system. Thus, the OET system could manipulate the particles with a wide range of sizes from 2 μm to 150 μm. Moreover, the manipulation velocity increased as the diameter of the particle increased. The maximum manipulation velocity was 4.04 μm/s. Meanwhile, the temperature of a large objective lens was higher than that of a small objective lens, because the concentration of light was stronger when the magnification of the objective lens was increased. For this OET system, the whole temperature was below 37.5 °C, which would not damage cells and other bioparticles. The velocity decreased with the decrease of light power. In addition, macrophages, *Euglena gracilis*, and spirulina were used as particles with different shapes for manipulation in the system. Finally, we demonstrated the versatile manipulation in the OET system, such as transporting particles in the maze, patterning particles into a “Hi” word or map outline of China, sorting particles from mixed solution, and storing particles at a specific zone. Moreover, the particle could be rotated for 360° on the bottom plane along with the vertical *z*-axis. These manipulation functions can be integrated into a single microfluidic chip of the OET system, depending on the flexible design of the light patterns. Meanwhile, the manipulation efficiency was not only related to optical patterns but also much related to the frequency, voltage, and the conductivity of the medium. The non-contact versatile manipulation reduces operating process and contamination. In future work, the simple optoelectronic tweezers system can be used to control non-contaminated cell interaction and micro/nano manipulation.

## Supplementary Materials

The following are available online at https://www.mdpi.com/2072-666X/12/3/271/s1, Video S1: Simulation of light spot pushed particle by OET. Video S2: Transportation of the microparticle via OET. Video S3: Patterning the microparticles via OET. Video S4: Sorting the microparticles via OET. Video S5: Storage of the microparticles via OET. Video S6: Rotating the microparticle via OET.
